# Changes in vitamin D status considering hemodilution factors in Japanese pregnant women according to trimester: A longitudinal survey

**DOI:** 10.1371/journal.pone.0239954

**Published:** 2020-10-02

**Authors:** Nobuko Takaoka, Keiko Nishida, Toshimi Sairenchi, Mitsumasa Umesawa, Rie Noguchi, Katsumi Someya, Gen Kobashi

**Affiliations:** 1 Department of Public Health, Dokkyo Medical University School of Medicine, Mibu, Japan; 2 Office of International Affairs, Center for International Cooperation, Dokkyo Medical University, Mibu, Japan; 3 Department of Obstetrics and Gynecology, Faculty of Medicine, University of Tsukuba, Tsukuba, Japan; 4 Dokkyo Medical University, Mibu, Japan; 5 Department of Obstetrics & Gynecology, Ibaraki Seinan Medical Center Hospital, Sashima, Japan; Charles P. Darby Children's Research Institute, 173 Ashley Avenue, Charleston, SC 29425, UNITED STATES

## Abstract

**Objectives:**

There have been no longitudinal surveys on the changes in 25-hydroxyvitamin D [25(OH)D] while considering hemodilution factors among pregnant Japanese women. Therefore, we examined 25(OH)D levels as well as red blood cell count (RBC), hemoglobin (Hb), and Hematocrit (Hct) at the three trimesters among pregnant Japanese women to determine the distribution of serum 25(OH)D levels and the influence of hemodilution.

**Design:**

This was a longitudinal study.

**Setting:**

The study was conducted at Ibaraki Seinan Medical Center Hospital in Japan.

**Participants:**

The participants comprised 50 women in the first trimester with singleton pregnancies and without any complications.

**Outcome measures:**

Participants were recruited from June to August 2018, and followed up till their postpartum period. Blood samples were collected at the first, second, and third trimesters, i.e., at 4–15, 16–27, and 28–39 weeks, respectively. 25(OH)D levels, RBC, Hb, and Hct were analyzed across the three trimesters.

**Results:**

Comparing the first, second, and third trimesters, 25(OH)D, RBC, and Hb were significantly declined in the second and third trimesters (p < 0.001). According to Spearman’s correlation coefficient with 25(OH)D and RCB, Hb, Hct, significant correlations were found between 25(OH)D and Hb (p < 0.001), as well as Hct (p < 0.001) in the third trimester.

**Conclusions:**

The present study had two major findings. First, it showed that the vitamin D status of most pregnant Japanese women were considered as vitamin D deficient. Second, the maternal serum 25(OH)D levels, RBC, Hb, and Hct of the pregnant women declined in the second and third trimesters. Thus we propose to have routine screening of vitamin D status of pregnant women, especially in the second trimester.

## Introduction

Previous studies have identified low maternal vitamin D level has already been recognized as a risk factor for various adverse perinatal outcomes, including preeclampsia [1–4], gestational diabetes mellitus [5, 6], and low neonatal birth weight [7]. A recent study reported that low maternal vitamin D level could be a risk factor for gestational anemia [8]. In addition, maternal serum 25-hydroxyvitamin D [25(OH)D] level is associated with bone, lung, and brain development of the fetus [9]. As the 25(OH)D level represents the vitamin D status, it is used as an indicator of vitamin D.

Further, the deficiency of maternal 25(OH)D during pregnancy can influence the later life of the offspring [9]. Maternal vitamin D deficiency at 18 weeks of pregnancy was associated with reduced lung function in children at the age of 6 years [10], language impairment at ages 5 and 10 [11], increasing eating disorder by the age of 20 [12]. These findings suggest that low maternal 25(OH)D level during pregnancy can be considered a common issue and a significant public health problem worldwide [13–15].

For the general Japanese population, the assessment criteria for vitamin D deficiency/insufficiency was proposed in 2017 by an expert panel supported by the Research Program of Intractable Diseases of the Japanese Ministry of Health, Labour and Welfare, Japanese Society for Bone and Mineral Research, and the Japan Endocrine Society [16]. The assessment criteria for vitamin D deficiency/insufficiency proposed by the expert panel was as follows: 25(OH)D equal to or above 30 ng/mL was considered to be vitamin D sufficient; 25(OH)D less than 30 ng/mL but not less than 20 ng/mL was considered to be vitamin D insufficient; 25(OH)D less than 20 ng/mL was considered to be vitamin D deficient. Low maternal 25(OH)D has been reported among Japanese pregnant women [17–23], even though the assessment criteria for vitamin D deficiency/insufficiency among pregnant women has not been defined yet. The proposed assessment criteria have mentioned that different criteria may be needed for pregnant women. In order to support the defining of appropriate criteria for the vitamin D status of Japanese women during pregnancy, studying the distribution of maternal serum concentration of 25(OH)D in the first, second, and third trimesters is necessary.

A previous cross-sectional sub-study [18] comprising pregnant Japanese women, carried out as an adjunct study of the Japan Environment and Children’s Study (JECS), and a longitudinal study determining the vitamin D level of Japanese pregnant women—the Saitama, Kobe, Yokohama Pregnant Cohort Study (SKY Study) [23]—indicated the 25(OH)D level during pregnancy. However, these studies did not consider the effects of plasma volume expansion due to pregnancy. Plasma volume increases in the first of weeks of pregnancy, with the steepest increase occurring during the second trimester, after which it continues to increase further in the third trimester [24]. During pregnancy, 25(OH)D level may be influenced by physiological hemodilution. Also, blood cell count (RBC), hemoglobin (Hb), and hematocrit (Hct) could be the indicators of hemodilution. Thus, we examined the 25(OH)D level as well as the red blood cell count (RBC), hemoglobin (Hb), and hematocrit (Hct) across all the three trimesters among pregnant Japanese women, in order to indicate the distribution of serum 25(OH)D level as well as the influence of hemodilution.

## Method

### Study design and participants

The present study was conducted as a longitudinal survey of pregnant women at the Ibaraki Seinan Medical Center Hospital in Japan, located N36.1°. Fifty women with singleton pregnancies who were in their first trimesters and without any complications were recruited from June to August 2018, and followed up until their postpartum period. Blood samples were collected during the first (4–15 weeks), second (16–27 weeks), and third (28–39 weeks) trimesters to examine vitamin D status across the trimesters. Simultaneously, RBC, Hb, and Hct were also measured.

Written informed consent was obtained from each subject. The study design and protocol was approved by the Bioethics Committee of Dokkyo Medical University (Daigaku 29010) and the Ethical Committee of Ibaraki Seinan Medical Center Hospital (April 12, 2018).

### Data collection

Blood samples were collected at the first, second, and third trimesters. Blood was drawn from each seated subject into a polyethylene terephalate tube with an accelerator, and another polyethylene terephalate tube with anticoagulant. Biochemical analysis of the blood was performed on serum samples at SRL (Tokyo, Japan). 25(OH)D was measured using 25OH-Vitamin D total-RIA-CT Kit (DIAsource ImmunoAssays S.A., Brabant Wallon, Belgium) and ARC-8080 γ counter (Hitachi Ltd, Tokyo, Japan). The measuring range was 4 to 99900000ng/mL. Complete blood count was measured using XE-2100 automated multiparameter hematology analyzer (Sysmex Corp, Hyogo, Japan).

Information on demographic variables, medical history, and lifestyle factors such as duration of nausea gravidarum, time spent walking or bicycling, hours of sleep per day, duration of sunlight exposure, use of skin protection from sunburn, and frequency of consuming food rich in Vitamin D was collected via a self-administered questionnaire. Clinical data, such as date and type of delivery, were collected from the electronic medical records of Ibaraki Seinan Medical Center Hospital.

### Statistical analysis

Distribution of 25(OH)D levels, RBC, Hb, and Hct were measured for each trimester. Univariate analysis was performed for 25(OH)D level, RBC, Hb, and Hct in the first, second, and third trimesters. To confirm normal distribution, the Kolmogorov-Smirnov test was used. As the resulting distribution was a non-normal distribution, Wilcoxon Signed-Rank Test was used to examine changes in 25(OH)D, while paired t-test was performed to examine changes in RBC, Hb, and Hct between the first and second, and the first and third trimesters. For determining associations between 25(OH)D and RBC, Hb, and Hct, Spearman’s correlation coefficient test was used. Fisher's exact test was used for determining association between 25(OH)D change from the first to second trimester and answers to questions on lifestyle factors. All statistical tests were conducted with SAS version 9.4 (SAS Institute, Inc., Cary, NC).

## Results

Table 1 indicates baseline characteristics of the study subjects. Participants were aged 18–40 years old (mean 31.0), 59% of the participants were primiparas, and 97.8% had term deliveries.

**Table 1:** Baseline characteristics of participants.

Participants, n	46*
Age, y, mean (min-max)	31.0 (10–40)
Primigravida, %	34.8
Primipara, %	50
Pre-pregnancy BMI (kg/m^2^) < 25%	65.2
Term delivery 37w ≤, %	97.8
Caesarian section, %	15.2
Birth weight < 2500 g, %	6.5
Postpartum hemorrhage up to 2 hours ≥ 500ml, %	23.9

* For the 1st trimester, blood was drawn form 50 participants; there were no baseline data for 4 participants because of their withdrawal from this study or transfer to other medical facilities.

**Fig 1:**
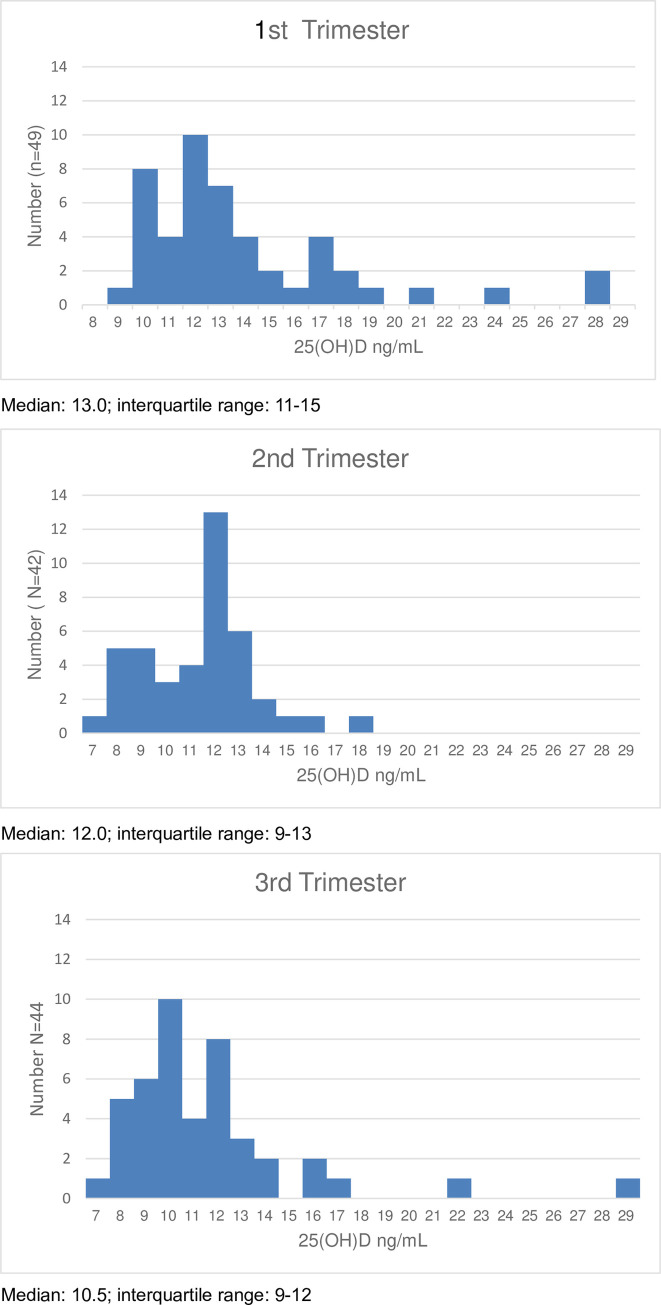
Distribution of the maternal serum 25(OH) D level across the three trimesters.

Fig 1 indicates distribution of serum 25(OH)D level at the first, second, and third trimesters. The Kolmogorov-Smirnov test rejected the normal distributions of the serum 25(OH)D level at the first, second, and third trimesters (p < 0.001, p < 0.010, and p < 0.010, respectively). The median and interquartile range of 25(OH)D in each trimester are indicated in the footnotes. Among the subject, percentage of vitamin D deficiency, 25(OH)D less than 20 ng/mL, in the first, second and third trimesters were 91.8%, 100%, 95.5%, respectably.

Table 2 indicates maternal serum 25(OH)D level across the three trimesters, stratified by parity, age, and pre-pregnancy body mass index (BMI) for all the three trimesters.

**Table 2:** Maternal serum 25(OH)D levels according to parity, age, and pre-pregnant BMI across three trimesters.

		25(OH)D ng/mL		
		1st trimester	2nd trimester	3rd trimester
Parity				
Primipara	N	23*	22	23
	median	12.0	11.0	11.0
	interquartile range	10.0–14.0	9.0–12.0	9.0–13.0
Multipara	N	23*	20	21
	median	13.0	12.0	10.0
	interquartile range	12.0–18.0	10.5–13.0	10.0–12.0
Age				
< 35	N	29	27	29
	median	13.0	12.0	10.0
	interquartile range	11.0–15.0	9.0–12.0	9.0–12.0
≥ 35	N	17	15	15
	median	12.0	12.0	12.0
	interquartile range	11.0–17.0	10.0–14.0	10.0–13.0
Pre-pregnant BMI				
< 25	N	30	27	28
	median	13.0	12.0	10.5
	interquartile range	12.0–17.0	10.0–13.0	9.0–12.5
≥ 25	N	16	15	16
	median	12.0	11.0	10.5
	interquartile range	10.5–13.5	9.0–13.0	10.0–12.0

* For the 1^st^ trimester blood were drawn form 50 participants, there were no baseline data for 4 participants because of their withdrawal from this study or transfer to other medical facilities.

Table 3 indicates the changes in 25(OH)D, RBC, Hb, and Hct across the trimesters. Serum 25(OH)D levels in the first, second, and third trimesters were 13.8±4.4ng/mL, 11.4±2.3ng/mL, and 11.5±3.9ng/mL, while Hct was 37.0±3.0%, 32.8±2.4%, and 33.5±2.5%, respectively.

According to the Wilcoxon Signed-Rank test results, the changes in 25(OH)D levels from the first to second trimester as well as from the first to third trimester were significantly negative (p < 0.001). Comparing the three trimesters using paired t-test, RBC, Hb, and Htc (p<0.001) were found to significantly decline. Maternal serum 25(OH)D, RBC, Hb, and Hct levels declined in the second and third trimesters.

**Table 3:** Change of 25(OH)D, RBC, Hb, and Hct according to trimester.

Trimester	1st	2nd	P 2nd vs 1st	3rd	P 3rd vs 1st
Mean ± SD					
25(OH)D, ng/mL	13.8 ± 4.4	11.4 ± 2.3	<0.001*	11.5 ± 4.0	<0.001*
Red blood cell, x10^4/μl	428 ± 34.5	370 ± 32.4	<0.001**	388 ± 36.0	<0.001**
Hemoglobin, g/dl	12.6 ± 1.0	11.0 ± 0.9	<0.001**	11.0 ± 1.0	<0.001**
Hematocrit, %	37.0 ± 3.0	32.8 ± 2.4	<0.001**	33.5 ± 2.5	<0.001**

*Wilcoxon Singed-Rank test

** Paired t-test.

Table 4 indicates Spearman’s correlation coefficient between 25(OH)D and RCB, Hb, Hct. A significant correlation was found in the third trimester between 25(OH)D and Hb (p < 0.001) as well as Hct (p < 0.001).

**Table 4:** Spearman’s correlation coefficient with 25(OH)D.

Trimester	1st	2nd	3rd
Spearman’s correlation coefficient			
Red blood cell, x10^4/μl	0.19	-0.04	0.26
Hemoglobin, g/dl	0.01	0.14	0.51*
Hematocrit, %	0.06	0.10	0.48*

*p < 0.05.

No association was found between the first and second trimester with respect to lifestyle factors, such as duration of nausea gravidarum, walking or bicycling, hours of sleep per day, duration of sunlight exposure, use of skin protection from sunburn, and frequency of consuming food rich in vitamin D (Table 5).

**Table 5:** Association of 25(OH)D level change from 1st to 2nd trimester with lifestyle.

Item		Change		*P value
		-16 ~ -2	-2 ~ +5	
Duration of nausea gravidarum, %	0 ~ 1 Week	33.3	20.8	0.81
	2 ~ 3 Weeks	11.1	12.5	
	4 ~ 7 Week	33.3	33.3	
	8 ~ Week	22.2	33.3	
Frequency of vomiting (Worst time), %	Several times/day	22.2	33.3	0.28
	Once/day	5.56	8.33	
	Once/few days	5.56	20.8	
	None	66.7	37.5	
Walking or bicycling 10 minutes per day before pregnancy, %	Yes	55.6	56.5	1.00
	No	44.4	43.5	
Walking or bicycling 10 minutes per day during pregnancy, %	Yes	50.0	34.8	0.53
	No	50.0	65.2	
Hours of sleep per day during pregnancy, %	<5h	0.0	16.7	0.36
	5h ~ 7h	55.6	45.8	
	7h ~ 9h	38.9	29.2	
	9h <	5.6	8.3	
Duration of sunlight exposure per day, %	1h <	55.6	56.5	1.00
	1h ≤ X < 2h	27.8	26.1	
	2h ≤ X < 4h	5.56	4.35	
	4h ≤	11.1	13.0	
Use of skin protection from sunburn, %	Yes	55.6	62.5	0.75
	No	44.4	37.5	
Frequency of eating food rich in vitamin D (Fish group), %	2 times/day	0.0	4.17	0.89
	Once/day	0.0	0.0	
	4~6 times/week	5.6	4.17	
	2~3 times/week	27.8	29.2	
	Once a week	38.9	29.2	
	Less than once a week	27.8	25.0	
	Not eating	0.0	8.3	
Frequency of eating food rich in vitamin D (Mushrooms group), %	2 times/day	0.0	0.0	0.15
	Once/day	11.1	0.0	
	4~6 times / week	16.7	8.3	
	2~3 times / week	27.8	29.2	
	Once a week	16.7	37.5	
	Less than once a week	27.8	12.5	
	Not eating	0.0	12.5	

* Fisher’s exact test.

## Discussion

The present study showed that vitamin D status of most pregnant women who participated in this survey were deficient. Furthermore, maternal serum 25(OH)D level, RBC, Hb, and Hct declined in the second and third trimesters. To the best of our knowledge, the present survey is the first to show these changes in serum 25(OH)D level and RBC, Hb, Hct of pregnant women in each trimester of pregnancy.

A systematic literature review [17] on the distribution of serum 25(OH)D among reproductive age Japanese women found that serum 25(OH)D level was especially low for perinatal women [21, 25, 26].

In the adjunct study of the JECS [18], it was indicated that in 1486 of the 2030 samples (73.2%), the serum 25(OH)D levels were less than 20ng/mL, suggesting that Japanese pregnant women had severe vitamin D deficiency status. The mean and standard deviation (SD) of the serum 25(OH)D level was 16.7±7.0ng/mL. These 2030 samples were collected over the four seasons in April, July, October, and January from 2012 to 2013.

In the SKY Study [23], 160 subjects were recruited from November 2010 to January 2011, and were followed up from the beginning of the first trimester. Blood samples were collected at 8–12, 22–24, and 32–34 weeks for the first, second, and third trimesters, respectively. The mean and SD of serum 25(OH)D in the first, second, and third trimesters were 9.2±3.6 ng/mL, 8.3±2.9 ng/mL, and 10.6±4.3 ng/mL, respectively. Regarding the 25(OH)D level, there were no changes across the trimesters. Comparing the present results to those of the SKY Study, 25(OH)D levels during pregnancy were found to be higher in all trimesters. In the present study, the mean and SD of the serum 25(OH)D in the first, second, and third trimesters were 13.8±4.4ng/mL, 11.4 ± 2.3ng/mL, and 11.5±3.9ng/mL, respectively. In addition, previous studies did not confirm the effect of hemodilution on 25(OH)D level.

Comparing among the adjunct study of JECS, SKY study and the present study, sample size was definitely different. In addition, in SKY and our study, subjects were recruited from one medical institution, in contrast, subjects of JECS study were from three prefectures. All study indicated 25(OH)D level of trimesters, however only our study indicated the timing of collecting the blood samples, which were in June to August for the first trimester, and October to December for the second trimester, and January to March for the third trimester.

The adjunct study of the JECS [18] found that when adjusted by the Dunnett correlation for multiple comparison, significant positive changes were found in the least square means for the maternal serum 25(OH)D level from the first to second trimester, 16.1ng/mL to 17.7ng/mL (p <0.001). However, our present results indicate significant negative changes in the mean of maternal serum 25(OH)D level from the first to the second trimester, 13.8ng/mL to 11.4ng/mL (p <0.001). Since JECS related study has no indication of season or period of blood sampling for the first and second semesters, seasonal effect was unclear.

The possible mechanism behind the changing level of maternal serum 25(OH)D with the progress of pregnancy might be explained by maternal plasma volume expansion, the function of placenta, vitamin D and calcium metabolism, and fetal development. Plasma volume begins increasing in the first week of pregnancy, with the steepest increase occurring during the second trimester. Using the plasma volume before conception or in non-pregnant controls as the reference value, the plasma volume increased by 0.18L (95% CI, 0.12–0.24L) (7.7% (95%CI, 5.1–10.3%)) in the first trimester and, plasma volume expanded by 0.57L (95% CI, 0.45–0.70L) (23.6% (95%CI, 18.4–28.8%)) in the second trimester of 15–21 weeks gestation. Also, at 22–28 weeks, the gestation plasma volume expanded by 0.98L (95% CI, 0.85–1.10L) (40.0% (95%CI, 35–45%)), and in the third trimester, it expanded by 1.13L (95% CI, 1.03–1.19L) (45.6% (95%CI, 43.0–48.1%)) [24]. The results of the present study implied that declining serum 25(OH)D, RBC, Hb, and Hct in the second trimester could be due to maternal hemedilution.

Regarding placenta function, development of the placenta is stabilized at the end of the first trimester. In the second trimester, fetal growth accelerates, which requires more maternal supply of nutrients such as vitamin D and calcium. Pregnancy induces dynamic changes in bone and calcium metabolism [27] as well as vitamin D metabolism [28]. For the latter, it is notable that only 25(OH)D readily crosses the placental tissue to the fetal compartment, while other forms of vitamin D do not [29], suggesting that the placenta is the major site of vitamin D metabolism in pregnancy [28].

Declining 25(OH)D during pregnancy may be influenced by not only plasma volume expansion, but also the increase of the necessary 25(OH)D for fetal development.

Regarding the association with lifestyle, the JECS related study [18] found that frequency of sunlight exposure and dietary intake of vitamin D were significantly associated with serum 25(OH)D levels, however, the SKY study [23] did not find significant associations between these variables during pregnancy. Additionally, in our study, no association was found between the change in the 25(OH)D level from the first to second trimester, and lifestyle factors such as duration of sunlight exposure, use of skin protection from sunburn, and frequency of consuming food rich in vitamin D. The analysis was not able to distinguish that obtained data was weather outlier. Further studies are necessary to examine the association between change in the 25(OH)D level from the first to the second trimester and lifestyle factors.

Our study indicated that the maternal serum 25(OH)D level in the second and third trimesters declined from that in the first trimester. It is imaginable that vitamin D requirement for the second and third trimesters would be higher. In order to define appropriate criteria for vitamin D status during pregnancy, further discussion is needed, including whether different criteria are necessary for each trimester or not.

The strength of the present study is that it shows the changes in not only the maternal serum 25(OH)D level, but also the RBC, Hb, and Hct across the three longitudinal points of pregnancy: the first, second, and third trimesters. In addition, all blood samples were measured using the same device, reagents, and quality control program.

Conversely, there are some limitations. First, because the sample size was relatively small, we were unable to confirm significant associations between lifestyle and 25(OH)D level. Second, seasonal effect on 25(OH)D has been well known. However, because the recruitment did not occur throughout the year, we could not adjust for seasonal effect. The SKY study [23] indicated similar to our result that 25(OH)D of the second trimester was lower than that of the first semester, even blood sample collection for the second trimester was estimated as between January to May. Third, serum 25(OH)D level was not measured by liquid chromatography-tandem mass spectrometry (LC-MS/MS) method which has become the gold standard for measuring 25(OH)D level.

## Conclusion

The present study had two major findings. First, it showed that the vitamin D status of most pregnant Japanese women were considered as vitamin D deficient. Second, the maternal serum 25(OH)D levels, RBC, Hb, and Hct of the pregnant women declined in the second and third trimesters. Thus we propose to have routine screening of vitamin D status of pregnant women, especially in the second trimester.
